# A Pilot Study on the Collection of Adverse Event Data from the Patient Using an Electronic Platform in a Cancer Clinical Trial Unit

**DOI:** 10.1007/s40801-024-00461-y

**Published:** 2024-11-02

**Authors:** Minna Grahvendy, Bena Brown, Laurelie R. Wishart

**Affiliations:** 1 Cancer Trials Unit, Princess Alexandra Hospital, Queensland Health, 199 Ipswich Rd, Woolloongabba, QLD 4102 Australia; 2 School of Health and Rehabilitation Sciences, The University of Queensland, Brisbane, Australia; 3 Southern Queensland Centre of Excellence in Aboriginal and Torres Strait Islander Primary Health Care, Metro South Hospital and Health Service, Brisbane, Australia; 4 School of Public Health, The University of Queensland, Brisbane, Australia; 5 Centre for Functioning and Health Research, Metro South Hospital and Health Service, Brisbane, Australia; 6 Centre for Applied Health Economics, School of Medicine and Dentistry, Griffith University, Brisbane, Australia; 7 Metro North Hospital and Health Service, Royal Brisbane and Women’s Hospital, Brisbane, Australia

## Abstract

**Background and Objective:**

Accurate and robust adverse event (AE) data collection is crucial in cancer clinical trials to ensure participant safety. Frameworks have been developed to facilitate the collection of AE data and now the traditional workflows are facing renewal to include patient-reported data, improving completeness of AE data. We explored one of these workflows in a cancer clinical trial unit.

**Methods:**

The study was a single-site study conducted at a tertiary hospital located in Australia. Patients consenting to a clinical trial were eligible for inclusion in this study. Participants used an electronic platform—My Health My Way (MHMW)—to report their symptomatic data weekly for 24 weeks. A symptom list was included within the platform, along with a free text field. Data reported via the platform was compared with data recorded in the patient’s medical chart. Time taken to compile data from each source was recorded, along with missing data points. Agreement between patient-reported data and data recorded in the medical notes was assessed using Kappa and Gwet’s AC_1_; time taken to compile data and missing data points were assessed using a Wilcoxon signed rank test.

**Results:**

Low agreement was found between patient- and clinician-reported data (− 0.482 and − 0.159 by Kappa and Gwet’s AC_1_ respectively). Only 127 (30%) of the total 428 AEs were reported by both MHMW and medical notes. Patients reported higher rates of symptoms from the symptom list, while clinicians reported higher rates of symptoms outside of the symptom list. Time taken to compile the data from MHMW was significantly less than that taken to review medical notes (2.19 min versus 5.73 min respectively; *P* <  0.001). There were significantly less missing data points from the MHMW data compared with the medical notes (1.4 versus 7.8; *P* < 0.001).

**Conclusions:**

This study confirms previous reports that patient- and clinician-reported adverse event data show low agreement. This study also shows that clinical trial sites could significantly reduce the work performed by research staff in the collection of adverse event data by implementing an electronic, patient-reported platform.

**Supplementary Information:**

The online version contains supplementary material available at 10.1007/s40801-024-00461-y.

## Key Points

**Table:** 

Collection of adverse event data directly from the patient via an electronic platform is possible.
Adverse event data collected directly from the patient shows low agreement with data recorded in the patient’s medical notes.
Utilising an electronic platform to collect patient-reported adverse event data can translate to time savings for staff of clinical trial sites.

## Introduction

The safety of participants in clinical trials is of paramount importance. Due to the atrocities of medical research that have occurred in the past, robust frameworks have been developed to protect the safety and rights of participants. One of these frameworks is the requirement to collect safety data on the intervention, as documented in the International Council for Harmonisation Guideline for Good Clinical Practice (ICH-GCP) [1]. This safety data is not only utilised to ensure the safety of participants during the trial but also informs safety labelling claims in the event the drug is approved for market [2]. For these reasons, it is crucial that accurate information on adverse events (AE) is collected during a clinical trial. To facilitate comparable AE data collection across cancer clinical trials, the National Cancer Institute (NCI) developed the Common Terminology Criteria for Adverse Events (CTCAE). This document allows clinicians to grade the severity of an AE according to five standardised grades; the current version (v5) includes 837 MedDRA terms including laboratory findings, observable and measurable events, and symptomatic events [3].

**Fig. 1:**
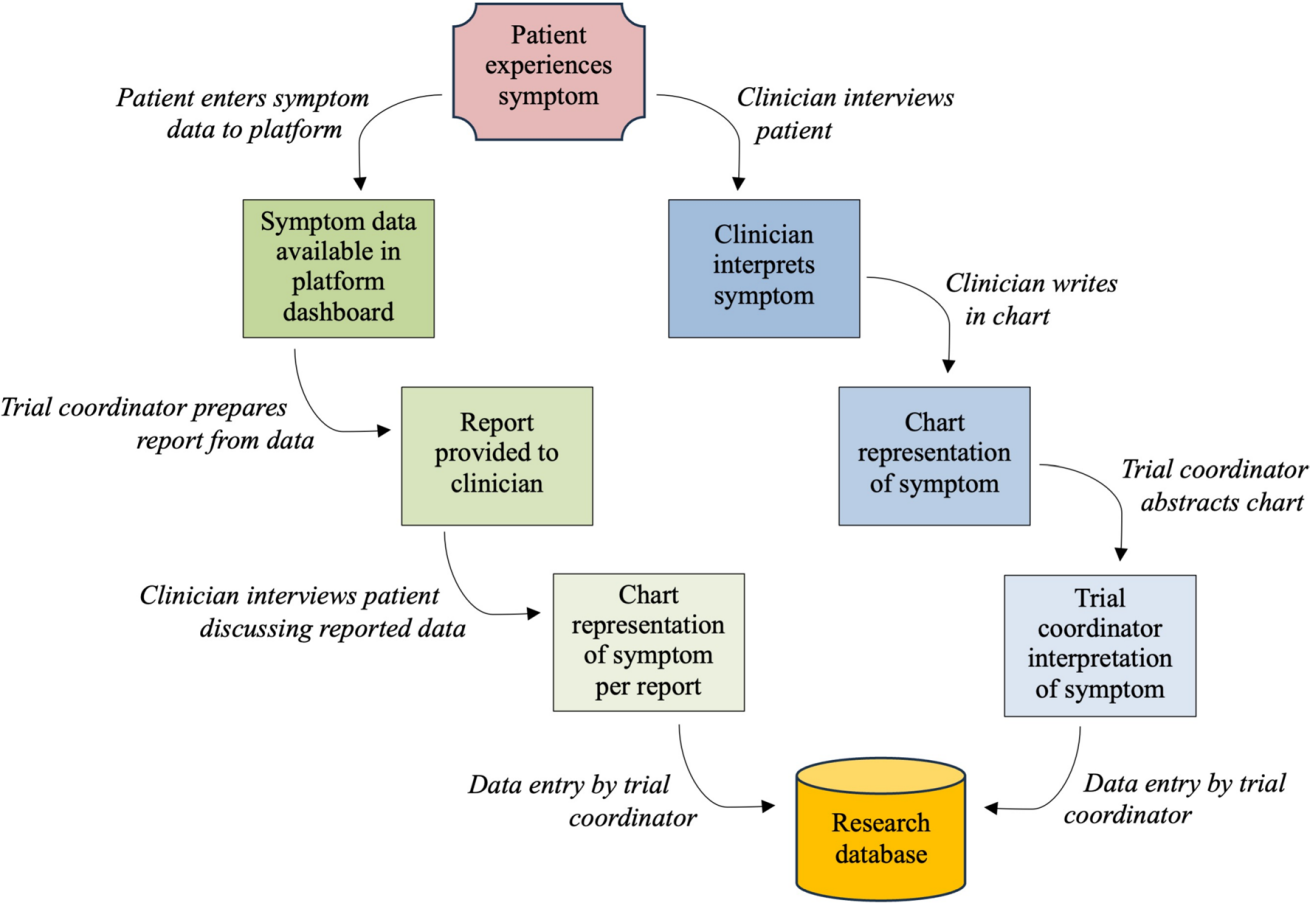
Current (blue) and proposed (green) workflows for reporting adverse events in clinical trials. Image adapted from Basch et al. [10, p. 3553]

Traditionally, AE data collection in cancer clinical trials occurs spontaneously when patients report signs and symptoms to their treating doctor during a consult. The doctor then grades the AE, assigns causality, and documents the AE in the patient’s medical chart. Following the consult, a member of the research team reviews the patient’s medical file, extracts the AE data and inputs the data into the sponsor’s research database (Fig. 1). Problems with this process are well-known, particularly regarding symptomatic AEs. In comparisons of clinician-reported and patient-reported AEs, clinicians have been found to underreport symptomatic AEs [4–6], while a study by Atkinson et al. [7] found that clinician interrater reliability is low. Compounding these inaccuracies is the effect of response shift and recall bias [8]; while symptom recall has been reported to be better than health-related quality of life recall, longer recall timeframes exacerbate recall bias [9].

Acknowledging the difficulties with the traditional workflow for collecting AE data, the clinical trial sector has been investigating the potential of collecting AE data directly from patients. Various proposals have been ventured as to how this data should be collected, analysed and reported [10–13], one option being to submit patient-reported AE data directly to the research database and reporting on this data as a standalone outcome measure. Another option, as described in Fig. 1 and reported in Kennedy et al. [13], is for patients to report symptomatic data to the clinical team using an electronic platform. These data are then provided to the clinician to discuss with the patient during review and subsequently entered into the research database as AE data as appropriate (reconciled report). In this workflow, the electronic platform functions as a data collection tool for patient-reported AE data that, following an interview between clinician and patient would be included in publications as part of the clinician-reported AE data. No consensus by the sector has yet been reached on how to best manage patient-reported AE data. Various studies [14–16] have investigated the feasibility of implementing patient-reported AE data collection systems in cancer clinical trials and have reported that such systems are feasible, that patient adherence with reporting is high (84–94%), and systems were well-received by site staff.

To facilitate this move to collecting patient-reported AE data, the NCI developed the patient-reported outcomes version of the CTCAE (PRO-CTCAE) [17]. This document includes plain language terms for the approximately 10% of items found in the CTCAE that correspond to symptomatic AEs. The PRO-CTCAE provides patients with the ability to rate symptoms on attributes of frequency, severity, and interference with daily life. A 5-point verbal descriptor scale is used to rate each attribute [18] and a composite grading algorithm has been developed to generate a single score from the attribute scores [19]. Currently, the PRO-CTCAE is the only validated tool designed for the collection of patient-reported AE data.

In addition to the deficiencies of the traditional workflow of AE data collection identified above, researchers in the clinical trial sector have investigated the burden placed on clinical trial sites through the traditional AE data collection process. Roche et al. [20] report an average of 18 min of research staff time is spent on conducting an AE assessment for a clinic visit, with an additional 35 min. taken to input AE data into the research database and document treatment. The clinical trial workforce shortage is an acknowledged issue in the sector [21–23] and particularly concerns clinical trial sites [21]. Staff shortages at trial sites limit the ability of the site to run clinical trials, which in turn limits treatment options available to patients, of which cancer clinical trials are an integral part of care pathways [21]. Streamlining workflows and utilising technology to lessen work burden in a stressed industry is therefore recognised as a priority.

In this study, we compared the use of an electronic, patient-reported platform with our current manual process for the collection of AE data for our clinical trial patients. The aims of this study are threefold: (1) compare data integrity and agreement between patients reporting AEs using an electronic platform versus clinician-reported AEs documented in medical notes, (2) report on patient adherence to self-reporting AE data and (3) compare trial coordinator work burden (in minutes) for AE data collection using the electronic platform versus the current manual process.

## Methods

### Participants and Study Design

Patients consenting to and found eligible to participate in an interventional clinical trial run by a cancer clinical trial unit at a tertiary hospital located in Australia were eligible for enrolment to this prospective, comparative, single-site feasibility study. Patients were recruited from the medical oncology and haematology outpatient clinics. Patients were eligible for this study if they had not yet started treatment on trial and had access to internet and a personal electronic device. Patients were followed on study for a period of 24 weeks, or until they discontinued treatment on their interventional clinical trial, or voluntarily withdrew from the study or clinical trial.

The study was approved by the Queensland Health Metro South Hospital and Health Service human research ethics committee (HREC/2021/QMS/73409) and all patients provided written, informed consent to participate.

### Survey and Administration

The platform used in this study was developed by researchers at the hospital where the study took place—My Health My Way (MHMW, previously ScreenIT) [24]. The platform is a person-centred, web-based, electronic platform used to capture common side effects experienced by patients with cancer, as well as data on physical, functional and psychosocial factors [25]. The performance of the MHMW platform has been assessed [24] and it was built using Qualtrics software [26].

The MHMW platform was adapted for use in this study by incorporating the PRO-CTCAE (v1.0) [17] survey along with date functionality [24]. Ten items from the English version of the PRO-CTCAE [27] were incorporated to a core symptom list, allowing patients to easily select these symptoms using radio buttons when present: nausea, vomiting, constipation, diarrhoea, dyspnoea, rash, paraesthesia, concentration, pain and fatigue. These items were chosen following surveying of senior trials staff to identify the ten most common symptoms experienced by participants enrolling in clinical trials across the two disciplines. Attribute scoring for each AE was included, and the composite scoring algorithm as published by Basch et al. [19] was automated in the background. Date functionality was added to the platform to allow participants to enter onset and resolution dates for symptoms; the platform tracked symptoms and prompted participants to confirm if previously reported symptoms were ongoing. Free text fields were available for participants to add additional symptoms not included in the above list (‘other’ symptoms).

A baseline symptom survey was completed prior to starting trial treatment, recall period for reporting was 7 days. Survey links were automated to be sent to participants via SMS or email on a weekly basis per patient preference, with reminders sent automatically after 24 h if the survey was not completed. As the platform is web-based, participants were able to complete the survey at a convenient time and location. The MHMW data were not shared with the treating clinician and participants were required to concurrently report all information on symptoms to their treating clinician during clinical review. These data were documented as per standard practice in the medical chart.

### Data Collection

#### Data Integrity and Agreement

Survey responses were collected and displayed in a dashboard format within the MHMW platform. To compare data integrity and agreement between patients reporting AEs using an electronic platform versus clinician-reported AEs documented in medical notes (aim 1), the following steps were completed. Once every 4 weeks a member of the research team reviewed the medical notes and compiled AE data in a tabular format containing available information on the AE term, clinician-reported CTCAE grade and start and stop dates of each reported AE. Following this, the same research member compiled a corresponding table from data presented on the MHMW dashboard. Medical note review was performed first to prevent bias due to prior knowledge of AEs reported via MHMW. CTCAE version 5.0 [3] was used in this study.

#### Adherence

Participant adherence to self-reporting AE data on the electronic platform (aim 2) was collected for each treatment cycle by recording the number of surveys delivered and completed.

#### Work Burden

To compare trial coordinator work burden (in minutes) for AE data collection using the electronic platform versus the current manual process (aim 3), time taken (in minutes) to compile the data from each source (MHMW electronic platform and medical notes) was measured. A count of missing data points (MHMW score or CTCAE grade, start date and stop date) was noted for each data collection method. One point was scored for each data point that was missing entirely, half a point was scored for data points that were noted descriptively but not specifically (e.g. the symptom started ‘yesterday’).

### Statistical Analysis

Descriptive statistical analysis was performed using IBM SPSS Statistics [28], while agreement statistical analysis was performed using R software [29] developed in RStudio [30]. Participant demographic data were analysed using descriptive statistics and the AE data (event term and score/grade) were compared using agreement statistics. Core symptoms were analysed separately to other symptoms. Vomiting was included as an 'other' symptom in the agreement analysis as there were only three instances of vomiting reported. Clinicians and patients were considered to be a single expert/informed rater for the purposes of the analysis.

Categorical variables were described using frequencies and percentages. To meet aim 1, MHMW versus medical notes detections and assessments were compared using cross-tabulation (confusion matrix) tables. For the binary categorical variables of interest, a Cohen’s kappa estimate was performed to assess the agreement in categorical variables between MHMW and medical notes. The judgement for the estimated kappa about the extent of agreement is given by Landis and Koch [31]: < 0, ‘no agreement’; 0–0.2, ‘slight agreement’; 0.2–0.4, ‘fair agreement’; 0.4–0.6, ‘moderate agreement’; 0.6–0.8, ‘substantial agreement’; 0.8–1.0, ‘almost perfect agreement’. Weighted kappa was used to assess the agreement between MHMW score and CTCAE grade. For the purposes of comparing MHMW score against CTCAE grade with statistical power, the MHMW ‘severe’ and ‘very severe’ scores were consolidated into ‘severe’. Additionally, where the medical notes did not report an event, the grade was set to ‘missing’ to prevent impact on the agreement.

Due to the nature of the study design the Gwet’s AC_1_ agreement coefficient was also derived per recommendation in Honda and Ohyama [32] and Reiter et al. [33]. Honda and Ohyama [32] provided supplementary R code but the R package irrCAC [34] was used instead. These analyses are largely exploratory, the study has not been powered for the approximate statistical tests. It does not take into account issues with multiple hypothesis testing. Any *P* values have not been corrected or adjusted. *P* values close to 0.05 have the potential of being a false-positive result.

To meet aim 2, participant adherence was assessed by calculating the proportion of surveys completed out of total surveys delivered. Overall participant adherence to the MHMW platform was analysed descriptively and adherence over time was analysed using the Kruskal–Wallis test. To meet aim 3, burden of work analysis consisted of a comparison of time taken (in minutes) to compile AE data as well as analysing missing data points via each method using the Wilcoxon signed-rank test.

## Results

### Characteristics of Study Participants

A total of 18 patients consented to the study; 6 participants were excluded from analysis due to noncompliance, having completed no MHMW surveys after the baseline survey. One participant was excluded from analysis due to remaining an inpatient during the entirety of their participation period and death prior to end of first cycle of treatment. Demographic data for the remaining cohort (*n* = 11) are described in Table 1. Average age at consent was not significantly different between the genders (*P* = 0.230) or chosen mode of survey delivery (*P* = 0.178).

**Table 1:** Participant demographic data

Characteristic	Number (SD, range) Total *n* = 11	Percent
Gender		
Male	7	63.6
Female	4	36.4
Other	0	0
Age at consent (years)		
All participants	61.8 (20.9, 19.8–81.5)	
Male	67.1 (18.9, 32.2–81.5)	
Female	52.6 (23.8, 19.8–76.6)	
Preference for survey delivery		
SMS	5	45.5
Email	6	54.4
Time on study		
Full study period completed	8	72.7
Full study period not completed	3	27.3
Reason for not completing study period		
Death	1	9.1
Discontinued clinical trial	1	9.1
Participant decision	1	9.1
Cancer type		
Hodgkin lymphoma	1	9.1
Amyloidosis	4	36.4
Non-small cell lung cancer	1	9.1
Squamous cell carcinoma	2	18.2
Multiple myeloma	1	9.1
Desmoid tumour	1	9.1
Intrahepatic cholangiocarcinoma	1	9.1

Patients were recruited from across ten clinical trials (one patient participated in two different clinical trials), of which eight were sponsored trials and two were collaborative group trials. Drug classes being investigated in the clinical trials were immune checkpoint inhibitors, monoclonal antibodies and small molecule inhibitors. One clinical trial was blinded with placebo, so it was not known if the participant was receiving the investigational product or placebo. The nature and number of AEs relative to the treatment protocol was not assessed as part of this study as it fell outside the scope, rather, the agreement and work burden between data collection methods was the primary aim, with an assessment of patient adherence to reporting.

### Adverse Event Terms Reported by MHMW Versus Medical Notes

A total of 428 AEs were reported across the MHMW platform or medical notes over the duration of the study. Key characteristics of these AEs are described in Table 2. AEs were more likely to occur during the first 8 weeks (time points 1 and 2); however, this is due to dropouts in later cycles with only eight participants (73%) completing the full study period. Core symptoms were reported at higher rates than other symptoms (271 (63%) and 157 (37%), respectively). Fatigue was the most common core symptom reported with 54 (13%) events and vomiting was the least common symptom reported with 3 (0.7%) events, followed by rash with 19 (4.4%) events. On average, MHMW reported 1.46 times more core symptoms compared with medical notes (230 versus 158 respectively; Supplementary Data Table 1), whereas medical notes reported 3.77 times more other symptoms compared with MHMW (132 versus 35 respectively; Supplementary Data Table 1). Only 35 of the 265 (13%) AEs reported by MHMW were reported using the free-text field.

**Table 2:** Key characteristics of adverse events reported in this study

Characteristic	No. of adverse events (*n* = 429)
Encounter	
Inpatient	26 (6%)
Outpatient	402 (94%)
Time point	
1	98 (23%)
2	82 (19%)
3	67 (16%)
4	60 (14%)
5	57 (13%)
6	64 (15%)
Core symptom	271 (63%)
Other symptom	157 (37%)
Core symptom	
Concentration	20 (4.7%)
Constipation	21 (4.9%)
Diarrhoea	26 (6.1%)
Fatigue	54 (13%)
Nausea	24 (5.6%)
Pain	33 (7.7%)
Paraesthesia	37 (8.6%)
Rash	19 (4.4%)
Short of breath	34 (7.9%)
Vomiting	3 (0.7%)

Only 127 (30%) of the total 428 AEs were reported by both MHMW and medical notes (Supplementary Data Table 1). Similar numbers of AEs were reported by medical notes and not participants (38%) as were reported by participants and not medical notes (32%). The overall agreement between MHMW and medical notes for the life of the study was analysed using Kappa and Gwet’s AC_1_ agreement coefficients, both tests indicated little-to-no consistent agreement (−0.482 and −0.159, respectively; Supplementary Data Table 2).

Agreement between MHMW and medical notes for each AE term was then assessed to determine if any AE term showed higher agreement (Supplementary Data Table 1). Concentration had the lowest agreement with 6 out of 20 (30%) events reported by both MHMW and medical notes, with MHMW reporting concentration at higher levels than medical notes. Constipation had the highest agreement with 17 out of 21 (81%) events being reported by both MHMW and medical notes. Kappa and Gwet’s AC_1_ were again used to assess agreement, vomiting was excluded from the analysis due to the low number of reported events. Constipation was removed from this analysis as there was no instance of it not being reported in medical notes, likewise, concentration was also excluded as there was no instance in which the term was reported in medical notes and not reported in MHMW. Kappa found little-to-no consistent agreement between MHMW and medical notes while Gwet’s AC_1_ agreement ranged from none to moderate (Supplementary Data Table 2). Rash showed the best agreement with Gwet’s AC_1_ (moderate), while fair agreement was found for paraesthesia, pain and shortness of breath.

No clear trends were found when agreement was assessed between MHMW and medical notes for AE terms over time; agreement ranged from 22% at time point 6 to 36% at time point 1 (Supplemental data Table 1). Kappa and Gwet’s AC_1_ show little-to-no consistent agreement across all timepoints (Supplementary Data Table 2).

### Adverse Event Scores Reported by MHMW Versus Medical Notes

Only 67 of the 428 (16%) AEs were scored/graded by both MHMW and in medical notes; 34% of these scores agreed overall over the life of the study (Supplementary Data Table 3). MHMW scored a total of 265 of the 428 (62%) events, whereas medical notes graded 143 (33%) events. Overall agreement of MHMW score and CTCAE grade was assessed by weighted Kappa and Gwet’s AC_1_; both analyses found slight agreement (0.086 and 0.068 respectively).

Further subgroup analysis of score/grade agreement by AE term or over time was not performed due to the low overall agreement. Cross table data for scores by AE term and over time can be seen in Supplementary Data Table 3. Grades documented in medical notes were more likely to be lesser than the corresponding severity score in MHMW.

### Participant Adherence

Total adherence to reporting via MHMW for all participants over the life of the study was 77.5% (range 44–100%). Kruskal–Wallis was used to assess for change in adherence over time and there was no significant change (*P* = 0.760; Supplementary Data Table 4).

### Work Burden

A total of 56 paired instances of timed comparisons of data collated from MHMW and corresponding medical notes were analysed (Supplementary Data Table 5). The average time taken to collate AE data from MHMW was 2.19 min (standard deviation [SD] 1.59 min) compared with 5.73 minutes (SD 6.01 min) for medical note review. The paired data were analysed using Wilcoxon signed rank test and a significant difference between the time taken to collate data from MHMW was seen compared with review of medical notes (*P* < 0.001).

The number of missing data points for the 56 paired instances of comparisons were analysed (Supplementary Data Table 5); the average missing data points for MHMW was 1.4 (SD 2.25) compared with 7.8 (SD 6.26) for medical note review. The paired data were analysed using Wilcoxon signed-rank test, and the difference in missing data points was significant (*P* < 0.001).

## Discussion

This pilot study lends evidence to the growing number of studies investigating the use of electronic, patient-reported platforms designed for the collection of AE data in cancer clinical trials. The current study found that agreement between AE terms reported by patients and clinicians was low, with patients reporting higher numbers of core symptom AEs, and clinicians reporting higher numbers of other symptom AEs. Patient scoring and clinician grading also found low agreement, with patients reporting higher scores. Patient adherence to reporting symptoms using MHMW was high over the life of the study, while MHMW provided significant reductions in work burden to research staff in the collection of AE data compared with the current manual process of reviewing medical notes.

Much thought has been published on the requirements of instruments used in the collection of patient-reported AE data. Basch et al. [35] provide these considerations in some detail, one of which being that careful consideration needs to be given to the set of symptoms included in any instrument designed for collection of AE data; a set of symptoms common to the disease or intervention should be selected. This can prove difficult for any cancer clinical trial site investigating the use of such an instrument in their standard workflow due to the range of diseases and drug classes being investigated at any one site. Even in this small study, patients were recruited from across two streams (oncology and haematology), seven diseases and three drug classes. Despite this, the ten core symptoms were reported at higher levels then all other symptoms combined, showing some success in the selection of these symptoms. However, patients were particularly reluctant to report other symptoms using the free text field included in the platform, indicating that better reporting rates of these symptoms could be achieved by including additional symptoms in the core symptom list. This notion is supported by Grahvendy et al. [36] where cancer clinical trial patients reported in their feedback on symptom reporting using an electronic platform that they prefer the ease of selecting symptoms from a list, rather than manually typing in symptoms.

A comprehensive symptom list in any patient-reported AE platform needs to be balanced with patient burden in utilising the platform, particularly for the clinical trial patient who already faces an increased burden in terms of additional trial-required assessments compared with standard care patients. Survey fatigue is an acknowledged problem in conducting clinical trials and can contribute to missed and incomplete data collection [37, 38]. So, while a comprehensive list could improve reporting accuracy, it should be specific to minimise burden. The low rates of patient-reporting using the free text field in this study also suggests that electronic, patient-reported AE platforms could not be expected to entirely replace medical note review for the collection of AE data. One potential solution to the conundrum of having a comprehensive yet specific symptom list at a clinical trial site that caters to diverse diseases and treatment classes would be to employ a platform that has a modular capability, with the capacity to create symptom lists specific to diseases and treatment class and assign patients accordingly.

Agreement between patient-reported and clinician-reported symptoms in this study was low, reflecting previously reported studies investigating concordance [5, 6, 39–43]. The results of this study further supports Basch et al. [44], wherein the authors report that symptoms that are more observable show higher agreement compared with those that are more subjective. This study found that symptoms such as rash, which is visible, and constipation, which is more easily quantifiable, were found to have higher agreement then concentration, which showed lowest agreement and is a purely subjective symptom. Comparison of patient scoring and clinician grading also showed little agreement in this study, reflecting previous reports [6, 41, 42]. It has, however, been acknowledged that patient scores and clinician grades are not expected to agree as they are differing measures [40, 45, 46]. Despite this, the patient score of their symptom is valuable data, providing information to the research team in real time on the patient’s status. If the platform includes an alert system, data on a patient’s worsening status can be transmitted to the treating team to facilitate early intervention and treatment of any worsening symptom.

Patient adherence to reporting symptoms via electronic platforms has been previously reported to be high (84–94%) [14–16]. Our study found lower rates of adherence (77.5%), though this could be due to the smaller study size. Adherence could potentially be improved if data collected by the platform was to be shared with the treating clinician and discussed with the patient during clinical review. A visible reinforcement to the patient that the data being reported by them is being used in their clinical care could motivate the patient to utilise the platform more consistently. Indeed, in a study by Grahvendy et al. [36], patient feedback on reporting symptomatic data reflected an expectation that these data will be shared with the treating team; similar sentiments have been published by Kennedy et al. [13].

A growing concern in the clinical trial sector is the worsening workforce shortage. Clinical trials provide valid treatment options for patients, and workforce shortages limit the ability of trial sites to run trials and provide these treatment options to patients. Our study showed that an electronic platform can significantly reduce the burden of AE data collection for research staff both in terms of collecting data and reducing the amount of missing data. Although the time taken to collect the data via the platform was significantly less than that taken to review medical notes, consideration should be given to the volume of data reported by patients utilising these platforms. Concerns have been shared by research staff that collection of patient-reported AE data could result in overburdening of the research team in both the volume of data collected and the generation of spurious data [13, 47]. Despite this concern, ICH-GCP requires that all AE data be reported. The significance of this is highlighted in a systematic review conducted by Sparano et al. [5], in which the researchers compared symptomatic AEs reported by patients and clinicians in randomised controlled trials in terms of if the data favoured the same treatment arm. They found that in the majority of studies (64.2%), there was discordance as to which arm was favoured by the AE data. In 50.2% of the studies, the patient-reported data favoured the experimental arm when the clinician-reported data did not. Likewise, in 14% of studies, the opposite was true. Complete AE data collection is critical to the safety of participants and accuracy of toxicity reporting of clinical trials, while efficient methods to collect these data are crucial to the operation of the clinical trial site.

The primary limitation encountered in this study was the small sample size which restricted the statistical analysis of the data. A small sample size was chosen to provide initial data on the implementation of an electronic, patient-reported AE platform in our cancer clinical trial unit. Despite the small sample size, significant results were detected, particularly in terms of the burden of work analysis. In this study, we did not investigate research staff or clinician feedback on the platform, primarily because platform and study data were not shared outside of the study’s research team, which forms another limitation in the assessment of the feasibility of MHMW. We now look forward to utilising the data from the current study in establishing and optimising a workflow to incorporate the MHMW platform in our AE data collection process.

## Conclusions

In this study, we explored the use of an electronic platform to collect AE data from the patient in a cancer clinical trial unit. The results of this study confirm previous reports that patient- and clinician-reported AE data show low agreement. We show that if provided with a comprehensive symptom list, patients can potentially report a higher volume of AEs compared with those documented in medical notes. Additionally, in line with previous reports, we found that cancer clinical trial patients are willing to report symptomatic data using an electronic platform with acceptable adherence rates. Finally, we show that implementation of an electronic, patient-reported AE platform can significantly reduce the work required by research staff in collecting this data. This study shows that implementing this platform in our unit has potential to improve our AE data, that our patients would be willing to utilise the platform and that there could be potential work efficiencies to the unit. Further work is required to obtain feedback from research and clinical staff on the performance of the platform.

## Supplementary Information

Below is the link to the electronic supplementary material.

Supplementary file1 (PDF 132 KB)

## Abbreviations

AE: Adverse event
ICH-GCP: International Council for Harmonisation Guideline for Good Clinical Practice
CTCAE: Common terminology criteria for adverse events
MHMW: My health my way
NCI: National Cancer Institute
PRO-CTCAE: Patient-reported outcomes version of the CTCAE

## Declarations

### Availability of data and material

The datasets generated during and/or analysed during the current study are available from the corresponding author on reasonable request.
