# Supplementary material for: A Pilot Study on the Collection of Adverse Event Data from the Patient Using an Electronic Platform in a Cancer Clinical Trial Unit
Source: Drugs Real World Outcomes. 2024 Nov 2;11(4):725–34. doi: 10.1007/s40801-024-00461-y (PMC11589020; doi:10.1007/s40801-024-00461-y)
Supplement: Supplementary file 1 — Supplementary file1 (PDF 132 KB) [file 40801_2024_461_MOESM1_ESM.pdf]

Article Title: A pilot study on the collection of adverse event data from the patient using an electronic platform in a cancer clinical trial unit

Journal: Drugs – Real World Outcomes

Authors: Minna Grahvendy, Bena Brown, Laurelie R Wishart

Corresponding Author:
Minna Grahvendy
Cancer Trials Unit, Princess Alexandra Hospital, Queensland Health, Brisbane, Queensland, Australia
minna.grahvendy@health.qld.gov.au

Supplemental data Table 1. Adverse event term agreement between MHMW and medical notes.

		Medical notes		
		No	Yes	Total
**Term**	**MHMW**			
Overall	No	0 (0%)	163 (38%)	163 (38%)
	Yes	138 (32%)	127 (30%)	265 (62%)
	Total	138 (32%)	290 (68%)	428 (100%)
Core symptom	No	0 (0%)	41 (15%)	41 (15%)
	Yes	113 (42%)	117 (43%)	230 (85%)
	Total	113 (42%)	158 (58%)	271 (100%)
Other symptom	No	0 (0%)	122 (78%)	122 (78%)
	Yes	25 (16%)	10 (6%)	35 (22%)
	Total	25 (16%)	132 (84%)	157 (100%)
Diarrhoea	No	0 (0%)	3 (12%)	3 (12%)
	Yes	13 (50%)	10 (38%)	23 (88%)
	Total	13 (50%)	13 (50%)	26 (100%)
Concentration	No	0 (0%)	0 (0%)	0 (0%)
	Yes	14 (70%)	6 (30%)	20 (100%)
	Total	14 (70%)	6 (30%)	20 (100%)
Constipation	No	0 (0%)	4 (19%)	4 (19%)
	Yes	0 (0%)	17 (81%)	17 (81%)
	Total	0 (0%)	21 (100%)	21 (100%)
Fatigue	No	0 (0%)	3 (6%)	3 (6%)
	Yes	32 (59%)	19 (35%)	51 (94%)
	Total	32 (59%)	22 (41%)	54 (100%)
Nausea	No	0 (0%)	2 (8%)	2 (8%)
	Yes	13 (54%)	9 (38%)	22 (92%)
	Total	13 (54%)	11 (46%)	24 (100%)
Paraesthesia	No	0 (0%)	4 (11%)	4 (11%)
	Yes	14 (38%)	19 (51%)	33 (89%)
	Total	14 (38%)	23 (62%)	37 (100%)
Pain	No	0 (0%)	7 (21%)	7 (21%)
	Yes	13 (39%)	13 (39%)	26 (79%)
	Total	13 (39%)	20 (60%)	33 (100%)
Rash	No	0 (0%)	10 (53%)	10 (53%)
	Yes	1 (5%)	8 (42%)	9 (47%)
	Total	1 (5%)	18 (95%)	19 (100%)
Short of breath	No	0 (0%)	7 (21%)	7 (21%)
	Yes	12 (35%)	15 (44%)	27 (79%)
	Total	12 (35%)	22 (65%)	34 (100%)
Vomiting	No	0 (0%)	1 (33%)	1 (33%)
	Yes	1 (33%)	1 (33%)	2 (67%)

	Total	1 (33%)	2 (67%)	3 (100%)
Timepoint 1	No	0 (0%)	32 (33%)	32 (33%)
	Yes	31 (32%)	35 (36%)	66 (67%)
	Total	31 (32%)	67 (68%)	98 (100%)
Timepoint 2	No	0 (0%)	31 (38%)	31 (38%)
	Yes	26 (32%)	25 (30%)	51 (62%)
	Total	26 (32%)	56 (68%)	82 (100%)
Timepoint 3	No	0 (0%)	26 (39%)	26 (39%)
	Yes	19 (28%)	22 (33%)	41 (61%)
	Total	19 (28%)	48 (72%)	67 (100%)
Timepoint 4	No	0 (0%)	23 (38%)	23 (38%)
	Yes	20 (33%)	17 (29%)	37 (62%)
	Total	20 (33%)	40 (67%)	60 (100%)
Timepoint 5	No	0 (0%)	24 (42%)	24 (42%)
	Yes	19 (33%)	14 (25%)	33 (58%)
	Total	19 (33%)	38 (67%)	57 (100%)
Timepoint 6	No	0 (0%)	27 (42%)	27 (42%)
	Yes	23 (36%)	14 (22%)	37 (58%)
	Total	23 (36%)	41 (64%)	64 (100%)

Supplemental data Table 2. Adverse event term agreement as assessed by Kappa and Gwet.

	**Kappa**		**Gwet**	
	**Coefficient value**	**Judgement**	**Coefficient value**	**Judgement**
**Term**				
Overall	-0.482	None	-0.159	None
Diarrhoea	-0.226	None	0.021	Slight
Fatigue	-0.037	None	-0.062	None
Nausea	-0.169	None	-0.096	None
Paraesthesia	-0.154	None	0.289	Fair
Pain	-0.173	None	0.234	Fair
Rash	-0.099	None	0.473	Moderate
Short of breath	-0.112	None	0.388	Fair
Timepoint 1	-0.474	None	-0.140	None
Timepoint 2	-0.451	None	-0.088	None
Timepoint 3	-0.463	None	-0.138	None
Timepoint 4	-0.431	None	-0.051	None
Timepoint 5	-0.519	None	-0.244	None
Timepoint 6	-0.559	None	-0.332	None

Supplemental data Table 3. Adverse event severity score and CTCAE grade agreement between MHMW and medical notes.

		**Medical notes**				
		Mild	Moderate	Severe	Unknown	Total
**Term**	**MHMW**					
Overall	Mild	15 (4%)	0 (0%)	0 (0%)	99 (23%)	114 (27%)
	Moderate	27 (6%)	8 (2%)	0 (0%)	63 (15%)	98 (23%)
	Severe	12 (3%)	5 (1%)	0 (0%)	36 (9%)	53 (13%)
	Unknown	56 (13%)	14 (3%)	6 (1%)	87 (20%)	163 (37%)
	Total	110 (26%)	27 (6%)	6 (1%)	285 (67%)	428 (100%)
Diarrhoea	Mild	3 (12%)	0 (0%)		12 (46%)	15 (58%)
	Moderate	1 (3.8%)	4 (15%)		1 (3.8%)	6 (23%)
	Severe	0 (0%)	1 (3.8%)		1 (3.8%)	2 (7.7%)
	Unknown	1 (3.8%)	0 (0%)		2 (7.7%)	3 (12%)

	Total	5 (19%)	5 (19%)		16 (62%)	26 (100%)
Concentration	Mild	0 (0%)	13 (65%)			13 (65%)
	Moderate	3 (15%)	3 (15%)			6 (30%)
	Severe	1 (5.0%)	0 (0%)			1 (5.0%)
	Total	4 (20%)	16 (80%)			20 (100%)
Constipation	Mild	3 (14%)	0 (0%)			3 (14%)
	Moderate	4 (19%)	1 (4.8%)			5 (24%)
	Severe	4 (19%)	5 (24%)			9 (43%)
	Unknown	3 (14%)	1 (4.8%)			4 (19%)
	Total	14 (67%)	7 (33%)			21 (100%)
Fatigue	Mild	1 (1.9%)	0 (0%)		17 (31%)	18 (33%)
	Moderate	6 (11%)	2 (3.7%)		13 (24%)	21 (39%)
	Severe	1 (1.9%)	2 (3.7%)		9 (17%)	12 (22%)
	Unknown	3 (5.6%)	0 (0%)		0 (0%)	3 (5.6%)
	Total	11 (20%)	4 (7.4%)		39 (72%)	54 (100%)
Nausea	Mild	2 (8.3%)	6 (25%)			8 (33%)
	Moderate	3 (13%)	9 (38%)			12 (50%)
	Severe	1 (4.2%)	1 (4.2%)			2 (8.3%)
	Unknown	1 (4.2%)	1 (4.2%)			2 (8.3%)
	Total	7 (29%)	17 (71%)			24 (100%)
Paraesthesia	Mild	1 (2.7%)	0 (0%)		14 (38%)	15 (41%)
	Moderate	6 (16%)	1 (2.7%)		6 (16%)	13 (35%)
	Severe	4 (11%)	0 (0%)		1 (2.7%)	5 (14%)
	Unknown	1 (2.7%)	0 (0%)		3 (8.1%)	4 (11%)
	Total	12 (32%)	1 (2.7%)		24 (65%)	37 (100%)
Pain	Moderate	2 (6.1%)	0 (0%)		14 (42%)	16 (48%)
	Severe	0 (0%)	0 (0%)		10 (30%)	10 (30%)
	Unknown	3 (9.1%)	1 (3.0%)		3 (9.1%)	7 (21%)
	Total	5 (15%)	1 (3.0%)		27 (82%)	33 (100%)
Rash	Mild	4 (21%)	0 (0%)		5 (26%)	9 (47%)
	Unknown	6 (32%)	1 (5.3%)		3 (16%)	10 (53%)
	Total	10 (53%)	1 (5.3%)		8 (42%)	19 (100%)
Short of breath	Mild	0 (0%)	0 (0%)		13 (38%)	13 (38%)
	Moderate	0 (0%)	1 (2.9%)		9 (26%)	10 (29%)
	Severe	1 (2.9%)	1 (2.9%)		2 (5.9%)	4 (12%)
	Unknown	1 (2.9%)	0 (0%)		6 (18%)	7 (21%)
	Total	2 (5.9%)	2 (5.9%)		30 (88%)	34 (100%)
Timepoint 1	Mild	4 (4.1%)	0 (0%)		27 (28%)	31 (32%)
	Moderate	4 (4.1%)	1 (1.0%)		10 (10%)	15 (15%)
	Severe	3 (3.1%)	0 (0%)		17 (17%)	20 (20%)
	Unknown	6 (6.1%)	1 (1.0%)		25 (26%)	32 (33%)
	Total	17 (17%)	2 (2.0%)		79 (81%)	98 (100%)
Timepoint 2	Mild	3 (3.7%)	0 (0%)	0 (0%)	20 (24%)	23 (28%)
	Moderate	9 (11%)	0 (0%)	0 (0%)	11 (13%)	20 (24%)
	Severe	0 (0%)	1 (1.2%)	0 (0%)	7 (8.5%)	8 (9.8%)
	Unknown	12 (15%)	1 (1.2%)	2 (2.4%)	16 (20%)	31 (38%)
	Total	24 (29%)	2 (2.4%)	2 (2.4%)	54 (66%)	82 (100%)
Timepoint 3	Mild	4 (6.0%)	0 (0%)	0 (0%)	13 (19%)	17 (25%)
	Moderate	4 (6.0%)	1 (1.5%)	0 (0%)	11 (16%)	16 (24%)
	Severe	3 (4.5%)	1 (1.5%)	0 (0%)	4 (6.0%)	8 (12%)
	Unknown	10 (15%)	3 (4.5%)	2 (3.0%)	11 (16%)	26 (39%)
	Total	21 (31%)	5 (7.5%)	2 (3.0%)	39 (58%)	67 (100%)
Timepoint 4	Mild	3 (4.9%)	0 (0%)		15 (25%)	18 (30%)
	Moderate	5 (8.2%)	1 (1.6%)		8 (13%)	14 (23%)
	Severe	3 (4.9%)	2 (3.3%)		0 (0%)	5 (8.2%)

	Unknown	11 (18%)	2 (3.3%)		11 (18%)	24 (39%)
	Total	22 (36%)	5 (8.2%)		34 (56%)	61 (100%)
Timepoint 5	Mild	0 (0%)	0 (0%)	0 (0%)	9 (16%)	9 (16%)
	Moderate	3 (5.3%)	2 (3.5%)	0 (0%)	14 (25%)	19 (33%)
	Severe	1 (1.8%)	1 (1.8%)	0 (0%)	3 (5.3%)	5 (8.8%)
	Unknown	8 (14%)	2 (3.5%)	2 (3.5%)	12 (21%)	24 (42%)
	Total	12 (21%)	5 (8.8%)	2 (3.5%)	38 (67%)	57 (100%)
Timepoint 6	Mild	1 (1.6%)	0 (0%)		15 (23%)	16 (25%)
	Moderate	2 (3.1%)	3 (4.7%)		9 (14%)	14 (22%)
	Severe	2 (3.1%)	0 (0%)		5 (7.8%)	7 (11%)
	Unknown	9 (14%)	5 (7.8%)		13 (20%)	27 (42%)
	Total	14 (22%)	8 (13%)		42 (66%)	64 (100%)

Supplemental data Table 4. Participant adherence (%) by cycle.

	**Timepoint 1**	**Timepoint 2**	**Timepoint 3**	**Timepoint 4**	**Timepoint 5**	**Timepoint 6**
Part.1	100	100	50	100	50	100
Part.2	100	100	100	100	100	100
Part.3	60	50	50	50	75	25
Part.4	100	100	100	75	25	RIP
Part.5	80	100	100	100	100	100
Part.6	100	100	100	75	100	100
Part.7	100	100	75	50	100	75
Part.8	60	25	25	WD	WD	WD
Part.9	100	100	75	WD	WD	WD
Part.10	80	50	50	50	50	50
Part.11	40	50	0	50	75	50

WD: withdrawn

Supplemental data Table 5. Paired data for MHMW and medical note review for time and missing data point analyses

**Timepoint**	**MHMW**		**Medical note review**	
	**Time (seconds)**	**No. of missing data points**	**Time (seconds)**	**No. of missing data points**
Part.1				
1	89	0.0	138	0.0
2	73	2.0	72	1.0
3	105	2.0	118	4.0
4	208	2.0	114	1.0
5	119	0.0	84	3.0
6	310	0.0	185	16.0
Part.2				
1	419	6.0	482	20.0
2	211	0.0	617	17.0
3	196	0.0	493	18.5
4	198	0.0	351	10.0
5	159	2.0	260	20.0
6	157	2.0	199	14.0
Part.3				
1	249	0.0	2018	25.5
2	98	0.0	906	19.0

3	70	0.0	522	11.0
4	35	0.0	336	5.0
5	132	4.0	241	5.0
6	109	0.0	427	4.0
Part.4				
1	370	12.0	237	9.0
2	233	6.0	231	13.0
3	195	0.0	365	6.0
4	144	2.0	229	5.0
Part.5				
1	139	2.0	1504	20.5
2	149	4.0	330	4.0
3	130	0.0	190	9.0
4	135	4.0	68	3.0
5	107	2.0	123	9.0
6	89	0.0	165	8.0
Part.6				
1	134	4.0	383	11.0
2	90	0.0	169	8.0
3	75	0.0	153	10.0
4	21	0.0	187	9.0
5	21	0.0	157	15.0
6	19	0.0	233	7.0
Part.7				
1	468	4.0	880	5.0
2	162	2.0	153	8.0
3	136	2.0	312	4.0
4	80	0.0	235	5.0
5	89	0.0	107	2.0
6	86	0.0	97	3.0
Part.8				
1	256	0.0	260	5.0
2	90	0.0	200	11.0
Part.9				
1	106	0.0	314	10.0
2	108	0.0	161	5.0
Part.10				
1	107	2.0	581	12.5
2	82	0.0	432	2.5
3	94	0.0	282	3.5
4	62	0.0	1189	11.5
5	40	2.0	770	4.0
6	118	6.0	203	3.5
Part.11				
1	108	0.0	298	2.0
2	50	2.0	48	0.0
3	0	0.0	152	0.0
4	8	0.0	131	0.0
5	80	2.0	85	0.0
6	25	0.0	67	0.0
